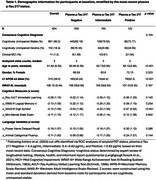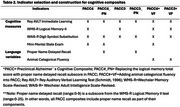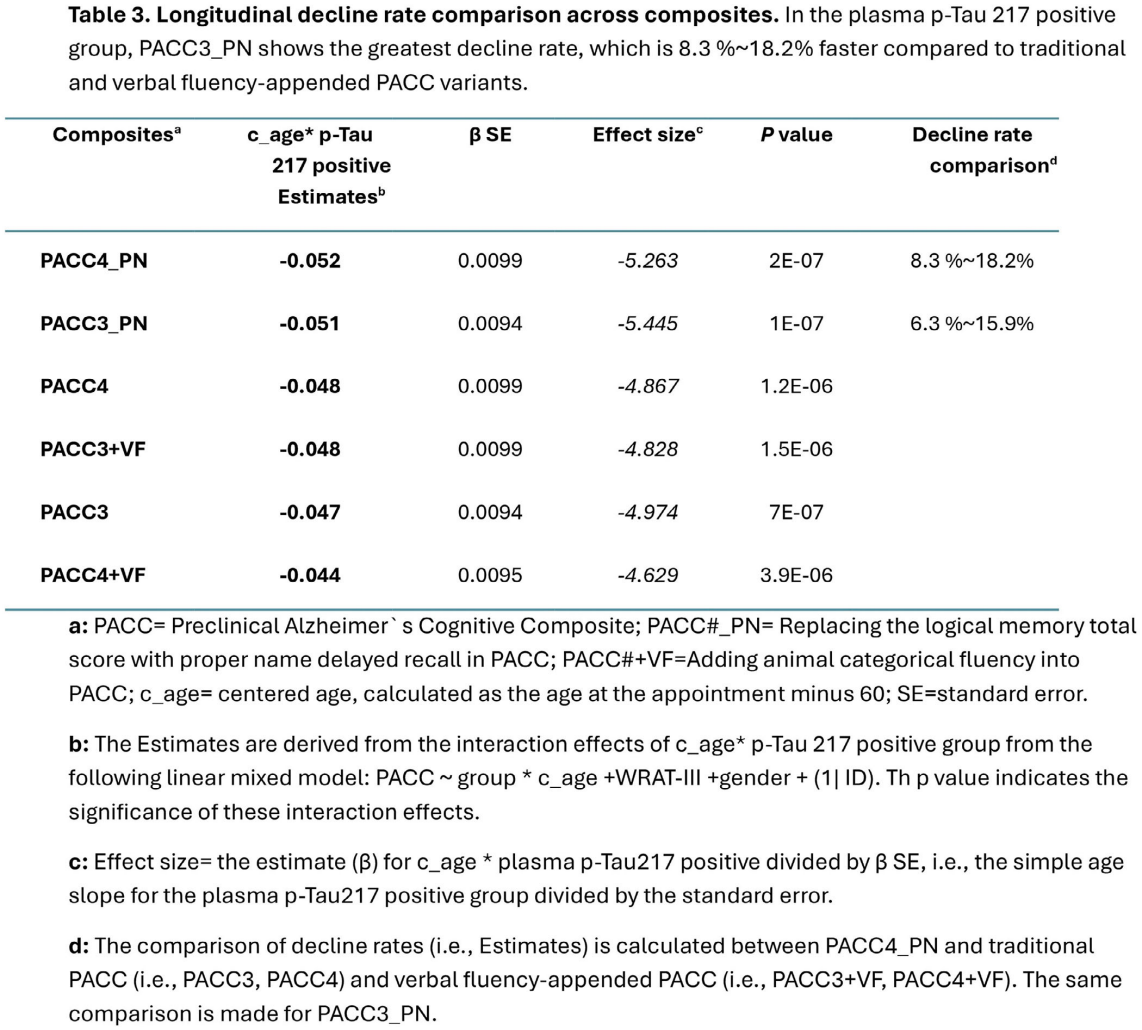# Enhanced Sensitivity to Elevated Plasma *p*‐Tau 217 with Proper Name Delayed Recall in the Preclinical Alzheimer's Cognitive Composite

**DOI:** 10.1002/alz70856_103547

**Published:** 2025-12-25

**Authors:** Deling He, Rebecca E. Langhough, Erin M. Jonaitis, Rachael E. Wilson, Bruce P Hermann, Sterling C Johnson, Kimberly D Mueller

**Affiliations:** ^1^ Department of Communication Sciences and Disorders, University of Wisconsin‐ Madison, Madison, WI, USA; ^2^ University of Wisconsin‐Madison School of Medicine and Public Health, Madison, WI, USA; ^3^ Wisconsin Alzheimer's Institute, University of Wisconsin School of Medicine and Public Health, Madison, WI, USA; ^4^ University of Wisconsin‐Madison, Department of Medicine, Madison, WI, USA; ^5^ Wisconsin Alzheimer's Disease Research Center, University of Wisconsin‐Madison School of Medicine and Public Health, Madison, WI, USA; ^6^ Wisconsin Alzheimer's Institute, University of Wisconsin‐Madison School of Medicine and Public Health, Madison, WI, USA; ^7^ Alzheimer's Disease Research Center, University of Wisconsin‐Madison School of Medicine and Public Health, Madison, WI, USA; ^8^ Wisconsin Alzheimer's Disease Research Center, University of Wisconsin‐Madison, School of Medicine and Public Health, Madison, WI, USA; ^9^ Department of Medicine, University of Wisconsin‐Madison School of Medicine and Public Health, Madison, WI, USA; ^10^ Wisconsin Alzheimer's Institute, University of Wisconsin School of Medicine and Public Health, Madison, WI, USA; ^11^ Department of Neurology, University of Wisconsin‐Madison School of Medicine and Public Health, Madison, WI, USA; ^12^ University of Wisconsin‐Madison, Madison, WI, USA; ^13^ Alzheimer's Disease Research Center, University of Wisconsin‐Madison, Madison, WI, USA; ^14^ Department of Communication Sciences and Disorders, University of Wisconsin‐Madison, Madison, WI, USA

## Abstract

**Background:**

The Preclinical Alzheimer's Cognitive Composite (PACC) is a vital tool for detecting early Alzheimer's disease (AD)‐related cognitive changes. Plasma *p*‐Tau217, a highly sensitive AD blood‐biomarker, reflects abnormal brain amyloid and tau accumulation. To increase the PACC's sensitivity to AD neuropathological change, we propose replacing the total score of Logical Memory with the proper name recall subscore⸺a preclinical language indicator highly sensitive to PET and CSF biomarkers.

**Method:**

We included 824 participants from the Wisconsin Registry for Alzheimer's Prevention (WRAP; Table 1; baseline dementia‐free). Using Ashton et al. (2024) cut‐offs for amyloid PET positivity, participants were grouped using their most recent plasma *p*‐Tau217 levels: negative (<0.4 pg/mL; *N* = 539), intermediate (0.4–0.63 pg/mL; *N* = 163), and positive (>0.63 pg/mL; *N* = 122). To construct PACC composites, we selected cognitive scores from published WRAP PACC (i.e., Rey AVLT immediate learning, Logical Memory II, Digit Symbol Substitution, MMSE), a novel lexical‐semantic indicator featuring proper name delayed recall, and a well‐established semantic memory test, animal categorical fluency. We constructed six PACC variants (Table 2). PACC3 and PACC4 include the first three and four tests listed, respectively. PACC#_PN replaces Logical Memory (range: 0‐50) with proper name recall (range: 0‐9), whereas PACC#+VF adds animal fluency to PACC3 and PACC4. Composite z‐scores (standardized using mean(sd) from all visits) were computed as the unweighted sum of z‐scores of contributing components (standardized using baseline mean(sd) of cognitively unimpaired‐stable subset). We evaluated longitudinal PACC decline via *p*‐Tau217 group*age interactions in linear mixed models, adjusting for gender and literacy. Sensitivity to *p*‐Tau217 across composites was compared using the simple age slope of the plasma *p*‐tau217 positive group.

**Result:**

Individuals with positive plasma *p*‐tau217 showed the fastest average rates of decline across cognitive composites (*p* <0.001); their declines in PACC4_PN and PACC3_PN were 8.3 %∼18.2% and 6.3 %∼15.9% faster than traditional and verbal fluency‐appended PACC (Table 3).

**Conclusion:**

Remarkably, substituting proper name delayed recall for logical memory total score improves sensitivity for tracking longitudinal cognitive decline related to AD blood‐biomarker *p*‐Tau217 group. Our study highlights a novel language measure that yielded composites more sensitive to AD‐related preclinical changes. Follow‐up analyses will investigate implications for screening and clinical trials.